# *cis*-Diammine(glycolato-κ^2^ *O* ^1^,*O* ^2^)platinum(II)

**DOI:** 10.1107/S1600536809049757

**Published:** 2009-11-28

**Authors:** Qing-Kun Wang, Shao-Ping Pu, Yan-Wei Cong, Yong-Nian Li, Chun-Fang Luan

**Affiliations:** a Kunming Institute of Precious Metals, Kunming 650106, People’s Republic of China; b Kunming IPM Pharmaceutical Co Ltd, Department of Materials, Kunming University of Science and Technology, Kunming 650106, People’s Republic of China; c Engineering School of Materials and Metallurgy, Kunming University of Science and Technology, Kunming 650093, People’s Republic of China

## Abstract

The reaction of *cis*-[Pt(NO_3_)_2_(NH_3_)_2_] and sodium glycolate yielded the title compound, [Pt(C_2_H_2_O_3_)(NH_3_)_2_]. The Pt^II^ atom, coordinated by two N atoms of ammine and two O atoms of the carboxyl­ate and oxido groups of the glycolate ligand, is in a square-planar environment. In the crystal structure, mol­ecules are connected by inter­molecular N—H⋯O hydrogen bonds, forming a three-dimensional network.

## Related literature

The title compound is a second-generation platinum derivative that has an anti­tumour activity comparable to that of cisplatin, one of the most effective anti-cancer drugs for testicular, lung, bladder and other carcinomas, but which is less toxic to the kidney, see: Inuyama *et al.* (1992 ▶); Kameyama *et al.* (1990 ▶); Noda *et al.* (1992 ▶); Taguchi *et al.* (1992 ▶); Yamamoto *et al.* (2000 ▶). For related structures, see: Yuge & Miyamoto (1998 ▶); Griffith *et al.* (2007 ▶).
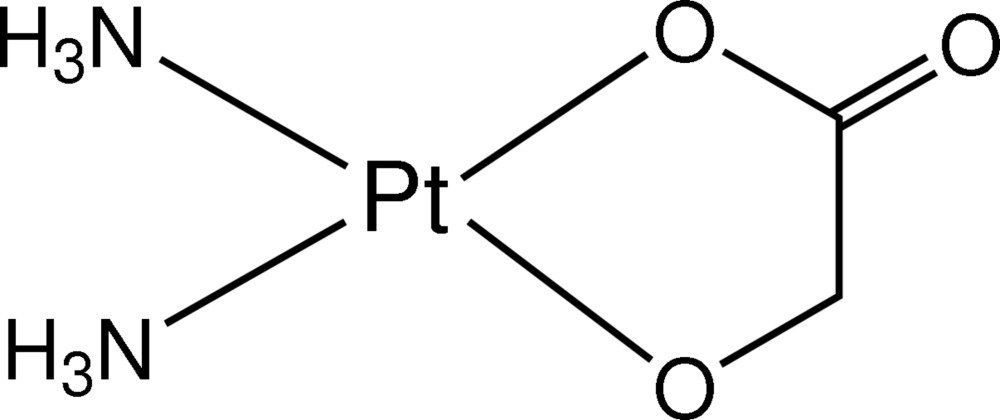


## Experimental

### 

#### Crystal data

[Pt(C_2_H_2_O_3_)(NH_3_)_2_]
*M* *_r_* = 303.19
Orthorhombic, 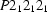

*a* = 5.6293 (6) Å
*b* = 7.2853 (8) Å
*c* = 14.1107 (16) Å
*V* = 578.70 (11) Å^3^
*Z* = 4
Mo *K*α radiation
μ = 24.17 mm^−1^
*T* = 298 K
0.24 × 0.12 × 0.10 mm

#### Data collection

Bruker APEXII CCD area-detector diffractometer
Absorption correction: multi-scan (*SADABS*; Sheldrick, 2002 ▶) *T*_min_ = 0.068, *T*_max_ = 0.196
3739 measured reflections
1354 independent reflections
1307 reflections with *I* > 2σ(*I*)
*R*_int_ = 0.034

#### Refinement

*R*[*F* ^2^ > 2σ(*F* ^2^)] = 0.020
*wR*(*F* ^2^) = 0.042
*S* = 0.99
1354 reflections
76 parameters
H-atom parameters constrained
Δρ_max_ = 1.24 e Å^−3^
Δρ_min_ = −1.10 e Å^−3^
Absolute structure: Flack (1983 ▶), 489 Friedel pairs
Flack parameter: 0.013 (17)

### 

Data collection: *APEX2* (Bruker, 2004 ▶); cell refinement: *SAINT* (Bruker, 2004 ▶); data reduction: *SAINT*; program(s) used to solve structure: *SHELXS97* (Sheldrick, 2008 ▶); program(s) used to refine structure: *SHELXL97* (Sheldrick, 2008 ▶); molecular graphics: *SHELXTL* (Sheldrick, 2008 ▶); software used to prepare material for publication: *SHELXTL*.

## Supplementary Material

Crystal structure: contains datablocks I, global. DOI: 10.1107/S1600536809049757/rn2056sup1.cif

Structure factors: contains datablocks I. DOI: 10.1107/S1600536809049757/rn2056Isup2.hkl

Additional supplementary materials:  crystallographic information; 3D view; checkCIF report

## Figures and Tables

**Table 1:** Hydrogen-bond geometry (Å, °)

*D*—H⋯*A*	*D*—H	H⋯*A*	*D*⋯*A*	*D*—H⋯*A*
N1—H1*C*⋯O2^i^	0.89	2.01	2.883 (8)	167
N1—H1*B*⋯O2^ii^	0.89	2.44	3.107 (7)	132
N1—H1*B*⋯O3^iii^	0.89	2.45	3.049 (7)	125
N1—H1*A*⋯O3^iv^	0.89	2.00	2.888 (7)	173
N2—H2*C*⋯O3^v^	0.89	2.32	3.108 (7)	147
N2—H2*B*⋯O2^ii^	0.89	2.21	3.014 (8)	150
N2—H2*A*⋯O3^iii^	0.89	2.26	3.010 (7)	142

Symmetry codes: (i) 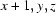; (ii) 
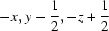
; (iii) 
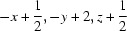
; (iv) 
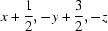
; (v) 
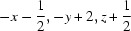
.

**Fig. 1.**
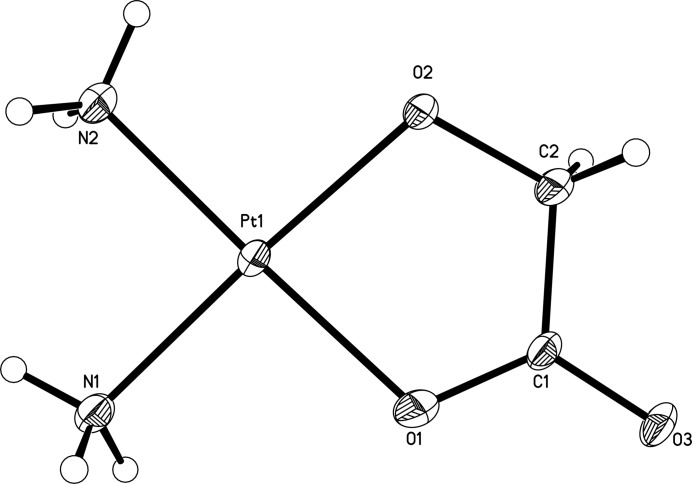
The molecular structure of title complex with the atomic labeling scheme. Displacement ellipsoids are drawn at the 30% probability level.

**Fig. 2.**
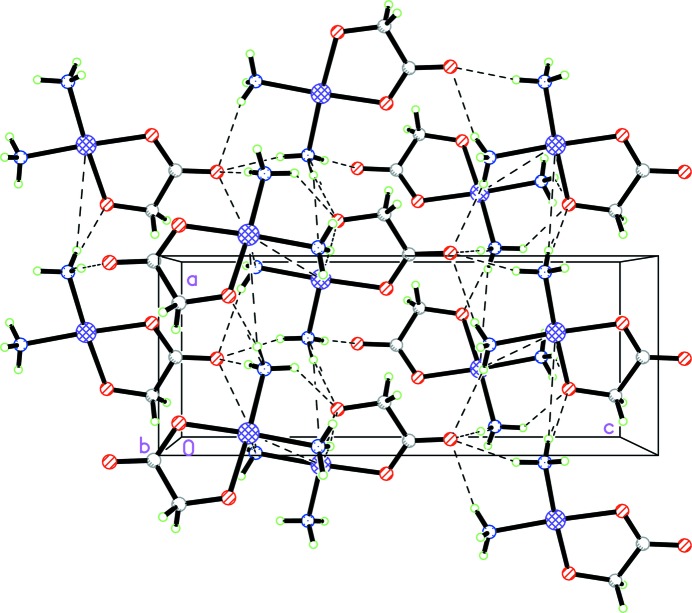
The crystal packing, showing the N—H···O hydrogen-bond network. Only the H atoms involved in hydrogen bonding are shown. Hydrogen bonds are shown as dashed lines.

## supplementary crystallographic information

### Comment

Cis-diamminedichloro-platinum(II) (cisplatin) is one of the most effective anti-cancer drugs for testicular, lung, bladder and other carcinomas. However, the clinical usefulness of this drug has frequently been limited by serious nephrotoxicity and gastrointestinal toxicity and the development of acquired resistance. In an attempt to overcome these drawbacks of cisplatin, numerous analogues have been prepared and evaluated in a search for alternative active agents. Among these compounds, the title compound, *cis*-diammine(glycolato-o,o')platinum(II), is a second-generation platinum derivative that has an antitumour activity comparable to cisplatin but is less toxic to the kidney (Kameyama *et al.*,1990), as seen in preclinical experiments. It produced promising response rates in phase II trials for treatment of squamous cell carcinoma arising from the head and neck (Inuyama *et al.*,1992), lung (Yamamoto *et al.*,2000), oesophagus (Taguchi *et al.*,1992), and uterine cervix (Noda *et al.*, 1992). For related structures see: (Yuge & Miyamoto, 1998; Griffith *et al.*, 2007) The compound forms a hydrogen-bonded structure (Fig. 2), in which one of the H atoms of ammonia serves as a donor to the O atom of the glycollate of an adjacent molecule and these hydrogen-bond interactions give rise to a three-dimensional network.

### Experimental

Cis-[Pt(NO_3_)_2_(NH_3_)_2_] (2.0 nmol) was dissolved in 50 ml water and sodium glycolate (2.0 mmol in 50 ml water) was added thereto. The mixture was adjusted to pH=7 with NaOH solution and stirred at 323k for 3 h. The solution was condensed at 313k under reduced pressure to 5 ml, then a yellow crystalline product was precipitated. The compound was crystallized from water to obtain crystals suitable for X-ray structure analysis.

### Refinement

All H atoms were initially located in a difference Fourier map. The H atoms bonded to carbon and nitrogen were placed at calculated positions (C—H = 0.97Å and N—H = 0.89 Å) and were included in the refinement in the riding model approximation, with *U*_iso_(H) = 1.2Ueq(C), *U*_iso_(H) = 1.5Ueq(N).

### Crystal data

**Table:**

[Pt(C_2_H_2_O_3_)(NH_3_)_2_]	*D*_x_ = 3.480 Mg m^−^^3^
*M**_r_* = 303.19	Mo *K*α radiation, λ = 0.71073 Å
Orthorhombic, *P*2_1_2_1_2_1_	Cell parameters from 1354 reflections
*a* = 5.6293 (6) Å	θ = 2.9–28.3°
*b* = 7.2853 (8) Å	µ = 24.17 mm^−^^1^
*c* = 14.1107 (16) Å	*T* = 298 K
*V* = 578.70 (11) Å^3^	Block, colourless
*Z* = 4	0.24 × 0.12 × 0.10 mm
*F*(000) = 544	

### Data collection

**Table:**

Bruker APEXII CCD area-detector diffractometer	1354 independent reflections
Radiation source: fine-focus sealed tube	1307 reflections with *I* > 2σ(*I*)
graphite	*R*_int_ = 0.034
phi and ω scans	θ_max_ = 28.3°, θ_min_ = 2.9°
Absorption correction: multi-scan (*SADABS*; Sheldrick, 2002)	*h* = −6→7
*T*_min_ = 0.068, *T*_max_ = 0.196	*k* = −9→9
3739 measured reflections	*l* = −17→18

### Refinement

**Table:**

Refinement on *F*^2^	Hydrogen site location: inferred from neighbouring sites
Least-squares matrix: full	H-atom parameters constrained
*R*[*F*^2^ > 2σ(*F*^2^)] = 0.020	*w* = 1/[σ^2^(*F*_o_^2^) + (0.0103*P*)^2^] where *P* = (*F*_o_^2^ + 2*F*_c_^2^)/3
*wR*(*F*^2^) = 0.042	(Δ/σ)_max_ = 0.001
*S* = 0.99	Δρ_max_ = 1.24 e Å^−^^3^
1354 reflections	Δρ_min_ = −1.10 e Å^−^^3^
76 parameters	Extinction correction: *SHELXL97* (Sheldrick, 2008), Fc^*^=kFc[1+0.001xFc^2^λ^3^/sin(2θ)]^-1/4^
0 restraints	Extinction coefficient: 0.0087 (3)
Primary atom site location: structure-invariant direct methods	Absolute structure: Flack (1983), 489 Friedel pairs
Secondary atom site location: difference Fourier map	Flack parameter: 0.013 (17)

### Special details

**Table:**

Geometry. All e.s.d.'s (except the e.s.d. in the dihedral angle between two l.s. planes) are estimated using the full covariance matrix. The cell e.s.d.'s are taken into account individually in the estimation of e.s.d.'s in distances, angles and torsion angles; correlations between e.s.d.'s in cell parameters are only used when they are defined by crystal symmetry. An approximate (isotropic) treatment of cell e.s.d.'s is used for estimating e.s.d.'s involving l.s. planes.
Refinement. Refinement of *F*^2^ against ALL reflections. The weighted *R*-factor *wR* and goodness of fit *S* are based on *F*^2^, conventional *R*-factors *R* are based on *F*, with *F* set to zero for negative *F*^2^. The threshold expression of *F*^2^ > σ(*F*^2^) is used only for calculating *R*-factors(gt) *etc*. and is not relevant to the choice of reflections for refinement. *R*-factors based on *F*^2^ are statistically about twice as large as those based on *F*, and *R*- factors based on ALL data will be even larger.

### Fractional atomic coordinates and isotropic or equivalent isotropic displacement parameters (Å^2^)

**Table:**

	*x*	*y*	*z*	*U*_iso_*/*U*_eq_	
Pt1	0.10734 (4)	0.96205 (3)	0.178777 (16)	0.01860 (9)	
N2	0.0429 (10)	0.9713 (9)	0.3193 (4)	0.0310 (12)	
H2A	0.1574	1.0347	0.3479	0.047*	
H2B	0.0392	0.8577	0.3424	0.047*	
H2C	−0.0964	1.0256	0.3296	0.047*	
N1	0.4267 (10)	0.8432 (8)	0.2082 (3)	0.0272 (13)	
H1A	0.4471	0.7457	0.1711	0.041*	
H1B	0.4298	0.8085	0.2686	0.041*	
H1C	0.5427	0.9237	0.1976	0.041*	
O1	0.1557 (8)	0.9447 (7)	0.0377 (3)	0.0308 (11)	
O2	−0.2005 (8)	1.0830 (7)	0.1425 (3)	0.0263 (11)	
C1	−0.0264 (12)	0.9948 (9)	−0.0099 (4)	0.0230 (15)	
C2	−0.2372 (12)	1.0639 (11)	0.0439 (5)	0.0332 (17)	
H2E	−0.2827	1.1823	0.0182	0.040*	
H2D	−0.3688	0.9803	0.0337	0.040*	
O3	−0.0334 (8)	0.9898 (7)	−0.0983 (3)	0.0288 (11)	

### Atomic displacement parameters (Å^2^)

**Table:**

	*U*^11^	*U*^22^	*U*^33^	*U*^12^	*U*^13^	*U*^23^
Pt1	0.02166 (13)	0.02149 (13)	0.01266 (12)	−0.00028 (10)	−0.00029 (9)	0.00010 (9)
N2	0.030 (3)	0.043 (3)	0.020 (3)	0.003 (2)	0.001 (2)	−0.003 (3)
N1	0.033 (3)	0.036 (3)	0.012 (2)	0.010 (3)	0.005 (2)	0.003 (2)
O1	0.028 (3)	0.046 (3)	0.018 (2)	0.004 (2)	0.0023 (19)	−0.006 (2)
O2	0.027 (2)	0.038 (3)	0.014 (2)	0.008 (2)	−0.0030 (18)	−0.0067 (19)
C1	0.027 (3)	0.024 (4)	0.018 (3)	−0.003 (2)	0.003 (2)	0.003 (2)
C2	0.032 (4)	0.052 (5)	0.016 (3)	0.011 (3)	−0.002 (3)	−0.004 (3)
O3	0.036 (3)	0.038 (3)	0.012 (2)	−0.002 (2)	−0.0002 (18)	−0.0005 (19)

### Geometric parameters (Å, °)

**Table:**

Pt1—O2	2.010 (5)	N1—H1B	0.8900
Pt1—O1	2.013 (4)	N1—H1C	0.8900
Pt1—N2	2.017 (5)	O1—C1	1.279 (8)
Pt1—N1	2.038 (5)	O2—C2	1.413 (7)
N2—H2A	0.8900	C1—O3	1.248 (8)
N2—H2B	0.8900	C1—C2	1.496 (9)
N2—H2C	0.8900	C2—H2E	0.9700
N1—H1A	0.8900	C2—H2D	0.9700
			
O2—Pt1—O1	83.82 (18)	Pt1—N1—H1C	109.5
O2—Pt1—N2	94.6 (2)	H1A—N1—H1C	109.5
O1—Pt1—N2	176.9 (2)	H1B—N1—H1C	109.5
O2—Pt1—N1	176.77 (19)	C1—O1—Pt1	113.2 (4)
O1—Pt1—N1	93.18 (18)	C2—O2—Pt1	109.5 (4)
N2—Pt1—N1	88.4 (2)	O3—C1—O1	122.8 (6)
Pt1—N2—H2A	109.5	O3—C1—C2	119.5 (6)
Pt1—N2—H2B	109.5	O1—C1—C2	117.7 (5)
H2A—N2—H2B	109.5	O2—C2—C1	114.7 (6)
Pt1—N2—H2C	109.5	O2—C2—H2E	108.6
H2A—N2—H2C	109.5	C1—C2—H2E	108.6
H2B—N2—H2C	109.5	O2—C2—H2D	108.6
Pt1—N1—H1A	109.5	C1—C2—H2D	108.6
Pt1—N1—H1B	109.5	H2E—C2—H2D	107.6
H1A—N1—H1B	109.5		
			
O2—Pt1—O1—C1	−6.9 (4)	Pt1—O1—C1—O3	−178.2 (5)
N2—Pt1—O1—C1	53 (5)	Pt1—O1—C1—C2	2.4 (8)
N1—Pt1—O1—C1	174.3 (5)	Pt1—O2—C2—C1	−11.1 (7)
O1—Pt1—O2—C2	9.7 (4)	O3—C1—C2—O2	−173.3 (6)
N2—Pt1—O2—C2	−167.7 (5)	O1—C1—C2—O2	6.1 (10)
N1—Pt1—O2—C2	31 (4)		

### Hydrogen-bond geometry (Å, °)

**Table:**

*D*—H···*A*	*D*—H	H···*A*	*D*···*A*	*D*—H···*A*
N1—H1C···O2^i^	0.89	2.01	2.883 (8)	167
N1—H1B···O2^ii^	0.89	2.44	3.107 (7)	132
N1—H1B···O3^iii^	0.89	2.45	3.049 (7)	125
N1—H1A···O3^iv^	0.89	2.00	2.888 (7)	173
N2—H2C···O3^v^	0.89	2.32	3.108 (7)	147
N2—H2B···O2^ii^	0.89	2.21	3.014 (8)	150
N2—H2A···O3^iii^	0.89	2.26	3.010 (7)	142

Symmetry codes: (i) *x*+1, *y*, *z*; (ii) −*x*, *y*−1/2, −*z*+1/2; (iii) −*x*+1/2, −*y*+2, *z*+1/2; (iv) *x*+1/2, −*y*+3/2, −*z*; (v) −*x*−1/2, −*y*+2, *z*+1/2.

## References

[bb1] Bruker (2004). *APEX2* and *SAINT*. Bruker AXS Inc., Madison, Wisconsin, USA.

[bb2] Flack, H. D. (1983). *Acta Cryst.* A**39**, 876–881.

[bb3] Griffith, D., Bergamo, A., Pin, S., Vadori, M., Helge, M. B., Sava, G. & Marmion, C. (2007). *Polyhedron*, **26**, 4697–4706.

[bb4] Inuyama, Y., Miyake, H., Horiuchi, M., Hayasaki, K., Komiyama, S. & Ota, K. (1992). *Gan To Kagaku Ryoho*, **19**, 871–877. 1605665

[bb5] Kameyama, Y., Okazaki, N., Nakagawa, M., Koshida, H., Nakamura, M. & Gemba, M. (1990). *Toxicol. Lett* **52**, 15–24. 10.1016/0378-4274(90)90161-e 2356567

[bb6] Noda, K., Ikeda, M., Yakushiji, M., Nishimura, H., Terashima, Y., Sasaki, H., Hata, T., Kuramoto, H., Tanaka, K., Takahashi, T., Hirabayashi, K., Yamabe, T. & Hatae, M. (1992). *Gan To Kagaku Ryoho*, **19**, 885–892. 1605666

[bb7] Sheldrick, G. M. (2002). *SADABS*. University of Göttingen, Germany.

[bb8] Sheldrick, G. M. (2008). *Acta Cryst.* A**64**, 112–122. 10.1107/S0108767307043930 18156677

[bb9] Taguchi, T., Wakui, A., Nabeya, K., Kurihara, M., Isono, K., Kakegawa, T. & Oka, K. (1992). *Gan To Kagaku Ryoho*, **19**, 483–488. 1558398

[bb10] Yamamoto, N., Tamura, T., Kurata, T., Yamamoto, N., Sekine, I., Kunitoh, H., Kodama, T. & Saijo, N. (2000). *Proc. Am. Soc. Clin. Oncol.* **19**, 203a (abstr 792).

[bb11] Yuge, H. & Miyamoto, T. K. (1998). *Inorg. Chim. Acta*, **297**, 105–110.

